# A patient with features of albright hereditory osteodystrophy and unusual neuropsychiatric findings without coding Gsalpha mutations

**DOI:** 10.1186/2251-6581-13-56

**Published:** 2014-05-22

**Authors:** Shirin Hasani-Ranjbar, Zahra Jouyandeh, Mahsa Mohammad Amoli, Akbar Soltani, Seyed Masoud Arzaghi

**Affiliations:** 1 Obesity & Eating Habits Research Center, Endocrinology and metabolism Cellular & Molecular Science Institute, Endocrinology & Metabolism research institute, Tehran University of Medical Sciences, Tehran, Iran; 2 Endocrinology and Metabolism Research center, Endocrinology & Metabolism research institute, Tehran University of Medical Sciences, Tehran, Iran; 3 Endocrinology & Metabolism Research Institute, 5th Floor, Shariati Hospital, North Kargar Ave., Tehran 14114, Iran

**Keywords:** Pseudohypoparathyroidism, GNAS mutation, Albright Hereditory osteodystrophy, Pseudopseudohypoparathyroidism

## Abstract

**Background:**

Pseudohypoparathyroidism(PHP) is a heterogeneous group of rare metabolic disorders characterized by hypocalcemia and hyperphosphatemia resulting from PTH resistance. Different forms of PHP have been reported based on biochemical and clinical manifestation and genetic findings. Most of these forms are caused by defects in GNAS, an imprinted gene locus with multiple subunits. We reported a 12- year- old girl with unusual clinical manifestations of Pseudopseudohypoparathyroidism(PPHP).

**Methods:**

After clinical and biochemical evaluations, the patients’ genomic DNA was isolated from peripheral blood leukocytes using salting out method. The whole coding sequences of GNAS gene including 13 exons were amplified by PCR. Quantitative PCR reactions were performed too.

**Findings:**

We described a 12- year- old girl with Albright Hereditory osteodystrophy (AHO) phenotype, poor school performance, some abnormal movements, TSH resistance with normal serum calcium and phosphorus levels and normal Gsα bioactivity with no mutation in GNAS exons. Unusual neuropsychiatric findings in this patient were compatible with Asperger syndrome.

**Conclusions:**

According to our findings this patient could not be categorized in any of PHP subgroups. Identifying of such individuals may be useful to discover different genetic patterns in pseudohypoparathyroidism and pseudopseudohypoparathyroidism. It is important to identify patients in whom PHP is caused by novel GNAS mutations, as careful investigations of these findings will likely further our knowledge of this complex and this unique disorder. In addition this case presented with unusual neuropsychiatric findings which has not been reported up to now.

## Background

Pseudohypoparathyroidism(PHP) is a heterogeneous group of rare metabolic disorders whose common feature is resistance to the parathyroid hormone which results to hypocalcemia and hyperphosphatemia [1,2]. Different forms of PHP are caused by mutations in those exons of GNAS encoding the α subunit [3]. Based on biochemical and clinical manifestation and genetic findings, variable forms of this disorder have been described.

Heterogeneous mutations located in GNAS exons which encode Gsα inherited from mother leads to PHP-Ia in which the characteristic phenotype appearance is Albright’s hereditary osteodystrophy(AHO), including obesity, short stature, heterotopis ossification, brachydactyly and/or mental retardation which has been described by Albright et al. for the first time in 1942 [4]. This form is also associated with other hormonal abnormalities, including hypothyroidism and hypogonadism [5]. Pseudopseudohypoparathyroidism(PPHP) a subgroup closely related to PHP-Ia have the typical features of AHO without any hormonal resistance [6] caused by heterozygous inactivating mutations in Gsα coding exons of GNAS which is inherited paternally [7]. In Type-Ib the resistance is restricted only to kidney, without AHO and other hormonal abnormalities. These patients have maternal microdeletions of STX16 which is associated with loss of methylation at GNAS exon A/B [1,3]. Another subtype called I-c is the same as type-Ia with no difference in clinical phenotype with in some patients, a mutation that affect the coupling of G protein to the PTH receptor [8,9]. Patients with PHP Type 2 do not have the physical appearance of AHO with a normal cAMP in response to PTH stimulation despite the inherent abnormality in calcium regulation [10,11]. However, recent reports have provided evidence that overlap can exist between the clinical features of different PHP subtypes [12,13]. Different subtypes of pseudohypoparathyroidism are shown in Table 1.

**Table 1:** Classification of pseudohypoparathyroidism with AHO phenotype

	**PTH resistance**	**Hormone resistance**	**Typical AHO phenotype**	**Gsα levels (%)**	**GNAS defects**
**PHP-Ia**	Yes	Yes	Yes	50	+ Gsα mutations
**PPHP**	No	No	Yes	50	± Gsα mutations
**Case**	No	Yes	Yes	100	- Gsα mutations

The psychiatric symptoms of pseudohypoparathyroidism have been reported to be related to Intracerebral calcification in basal ganglia [14]. Moderately severe to entirely normal school performance and moderate degrees of mental retardation has been reported in patients withPHP-Ia but not in PHP-Ib [15,16]. Seizure, tetany and laryngeal stridor may also occur in PHP-Ia because of hypocalcemia [17].

Here we present a case with Albright’s hereditary osteodystrophy phenotype, with normal calcium, phosphate and PTH levels, in which neurological symptoms, resistance to other hormones and negative GNAS gene mutations were unique findings.

## Patients and methods

### Mutation analysis of the GNAS gene

The patients’ genomic DNA was isolated from peripheral blood leukocytes using salting out method. The whole coding sequences of GNAS gene including 13 exons were amplified by PCR according to the assay described before [18].

### Molecular analysis

After collection of heparinized venous blood, lymphocyte separation was performed using density gradient ficoll (1.077) and total RNA was extracted using Tripure Isolation Reagent (Roche Applied Science), according to the manufacturer’s protocol. One μg aliquot of total RNA from patients sample and 3 normal control samples were reverse transcribed into single-stranded cDNA using random hexanucleotides primers and expand reverse transcriptase (Roche Applied Science). cDNA for sample was subjected to quantitative real-time PCR using the following primers for HPRT internal quantitative control forward 5′-CCTGGCGTCGTGATTAGTGAT-3′, reverse 5′- AGACGTTCAGTCCTGTCCATAA-3′ and GNAS, Forward 5′- TGTACAAGCAGTTAATCACCCACCA -3′ and reverse 5′- TCTGTAGGCCGCCTTAAGCTTTC -3′ primers.

### Quantitative PCR

For evaluation of Gs alpha gene quantitative PCR reactions were performed in 25 ul reaction mixtures containing 250 ng cDNA, 10 ml Takara Real-Time™ SYBR Green/ROX PCR Master, primer pairs and nuclease-free water to 20 ml. Each biological replicate was run in duplicate on an ABI Step One quantitative PCR system. Thermocycling conditions consisted of an initial polymerase activation step at 95°C for 10 min, followed by 40 cycles at 95°C for 5 s and 60°C for 30 s. Afterwards, melting curves were generated to confirm a single gene-specific peak and to detect primer dimmer formation.

## Case presentation

### Patient

**Figure 1:**
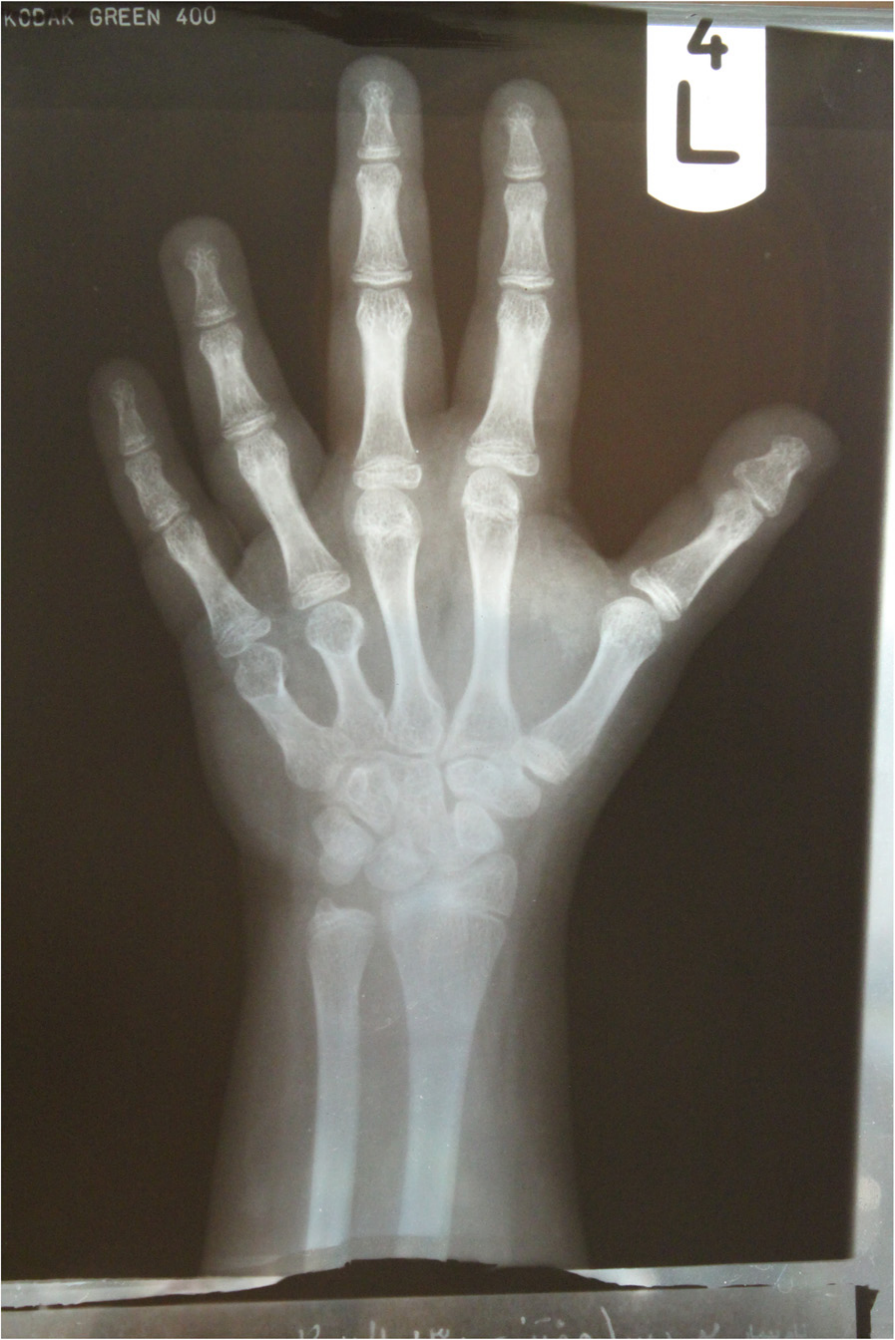
**Left hand x-ray of case.** Short 4^th^ & 5^th^ metacarpal.

A 12- year- old girl whom parents were distant relatives was referred to our clinic for evaluation of short stature and poor school performance. Her past medical history was positive for post term delivery (2 weeks delay) with cesarean section and meconium aspiration, poor feeding, severe high arced palate in infancy and a history of redden face while crying, amblyopia at the age of 5 year old, poor school performance and attention deficit (concentration disturbance). She showed some abnormal repetitive verbal and physical movements. Physical examination revealed obesity with short neck, round face and brown spots on her skin. Her height was 127 cm (below the third percentile) and her weight was 50 kg (body mass index > 97^th^ percentile). Mother’s height was 158 cm and father’s height was 172 cm. Both hands appeared short, with markedly short fourth and fifth metacarpals. Thyroid physical exam showed a 15–20 gr, normal, not firm gland without any palpable nodules. Laboratory data revealed normal serum calcium (9.5 mg/dl), phosphorus (5.6 mg/dl) and intact PTH (10 pg/ml). Serum TSH was 11 μU/ml. Her puberty showed normal timing (menarche at 11 y and 6 months). All laboratory tests are shown in Table 2.Radiography of the left hand revealed markedly short fourth and fifth metacarps (Figure 1). Bone age was compatible with her age. Electromyography (EMG) and Nerve conduction velocity (NCV) tests of four limbs were normal. Echocardiography showed an ejection fraction of 60%, other cardiac components were normal.

**Table 2:** Clinical and laboratory findings of the patient

**Variable**	**Patient**	**Normal range**
**Height (cm)**	**127**	**Below the 3th percentile**
**Weight (kg)**	**50**	**Above 97**^**th **^**percentile**
**Calcium (mg/dl)**	**9.5**	**8.5-10.5**
**Phosphate (mg/dl)**	**5.6**	**3.6-5.8**
**PTH (pg/ml)**	**10**	**10-62**
**TSH (μu/ml)**	**11**	**0.5-4**
**Anti-TPO Ab**	**Negative**	**<15**

Karyotype analysis on peripheral lymphocytes was 46, XX as a normal female chromosomal pattern with no chromosomal defect. We analyzed the 13 GNAS exons encoding Gsα in genomic DNA and no mutations or alternations could be identified. Gsα mRNA level, measured by qRt-PCR using primers at exons 2 an 11, showed that the level was not different from the controls. The subject and her family gave informed consent to the study.

### Neuropsychiatric findings

She had a febrile seizure at 1.5 years old and in early childhood recurrent generalized Tonic-clonic seizures with Abnormal EEG (frequent paroxysmal dysrhythmia) which treated up to now with provision of neurologist. Stereotyped and repetitive motor mannerisms (hand flapping) were present during infancy and early childhood as parents registered. Severe impairment in social interactions as failure to develop age-appropriate peer relationship and severe communication problems with family members especially with mother are present. Beside, the patient had ritualistic behaviors and compulsions (washing and repeating) which significantly impaired her functioning and proposed co-morbid obsessive-compulsive disorder. Academic failure was present from elementary school stage and total intellectual score using Wechsler intelligence scale was 68 and suggests mild mental retardation. Episodes of binge eating behavior present and patient was obviously obese. Magnetic Resonance Imaging (MRI) revealed bilateral ventricles enlargement due to Cavum Septum Pellucidum.

## Discussion

In this report, we present a patient with short stature and poor school performance, with elevated TSH level diagnosed as hypothyroidism. The patient had normal calcium and phosphate levels with no resistance to PTH hormone, which did not lead to suspicion of PHP-Ia or Ib. We found a short stature male in father’s family (his uncle) and a short stature member (grandfather) in mother’s family; however we have report of AHO phenotype in 2 cousins of her, which suggest the paternally inherited pattern of the disease.

These data and her laboratorial profile were compatible with PPHP [7,19]. The patient had normal serum concentrations of calcium and phosphate for his age, and just his elevated levels of TSH and his mother’s background was compatible with the diagnosis of PHP-Ia [17]. Mariana Tenorio Antunes Reis et al. [20] have also reported an infant boy with AHO phenotype, normal calcium and phosphate levels, whose mother was diagnosed as PHP-Ia. The functional analysis of GNAS exons showed a novel heterozygous missense mutation in exon 5 of GNAS. They suggest that AHO phenotype and normocalcemia does not always mean PPHP, especially when the allele is maternally inherited.

To investigate this possibility, the GNAS coding region of patient was studied. There was no typical mutation in GNAS coding region and the Gsα mRNA level did not appear to be deminished, which is compatible with Gsα pattern in PHP-Ic [9]. It should be noted that our qRT-PCR analysis of Gs-alpha mRNA was performed by generating an amplicon consisting of the region encoded by exons 2 through 11. Thus, given that certain other GNAS transcripts share this region, the level of Gs-alpha mRNA determined in our experiments may not entirely reflect the expression of Gs-alpha transcript in case of any signficant changes being observed in the level of expression. Sussane Thiele et al. described 5 patients diagnosed as PHP-Ic in whom they found TSH and PTH resistance, with normal Gsα activity and inherited maternal inactivating mutations in GNAS exons [21]. Linglart et al. [22] in a study on genotype, phenotype relationship on the maternal transmission of the hormonal resistance did not observe any relationship between the erythrocyte Gsα activity and the phenotype which may justify our findings in this patient.

This patient has high TSH and low anti TPO level which leads to hypothyroidism. Joaquin Lado-Abeal et al. [23] reported a family with congenital hypothyroidism due to maternal inherited loss-of-function mutation of TSH receptor and AHO phenotype in two children of family due to a paternal inherited mutation of GNAS,G alpha protein subunit. This study suggests that our subject may have the same inheritance pattern which leads to TSH resistance and AHO phenotype, although we do not have enough evidence to prove this hypothesis. Also studies have shown that Gsα has a tissue specific manner, as YU S et al. showed a mild PTH resistance despite normal TSH and thyroid hormone levels in a mice because of imprinted gene in renal tubules but not in other tissues [24]. A justification for some unusual and discordant findings in PHP patients which has been confirmed by other studies recently [25,26] may explain the hormonal resistance pattern in this case.

These unusual findings make a difficulty in net diagnosis for this case. This patient did not have PTH resistance which cannot be classified as type Ia or Ib and at the same time GNAS mutation was negative. Izraeli et al. also reported a new variant of the syndrome, affecting 5 individuals in a 3 generation family with AHO, normal Gs activity and hypothyroidism [27]. Giovanna Mantovani and colleges in a study on 40 patients have detected an absence of mutations in Gsα-coding GNAS exons although they had the presence of PTH resistance [12]. Due to Gsα inheritance pattern, it is suggested that PHP-Ia results from a heterozygous inactivating mutation at GNAS inherited from maternal allele while PPHP is inherited by paternal alleles [7]. In this case the paternally inheritance pattern, with normal Gsα activity and resistance to TSH hormone make a discordance to categorize this patient as PHP-Ia, Ic or PPHP. Although we did not do genome sequencing to identify specific GNAS mutations in this patient, Linglart et al. suggest that western blot could be used to assess Gsα protein expression. They have report three patients with PHP-Ia phenotype without any GNAS mutations that hypothesize that normal Gsα protein expression and the absence of a mutation in the coding regions makes the presence of a GNAS1 molecular defect unlikely [22]. Davids et al. proposed that an alternative etiology for PPHP is the deletion of *STK25*, inhibiting G protein signaling by a mechanism different from mutation of the Gsα gene which may account for the PPHP condition in the small group of patients with normal Gsα who still experience the AHO phenotype [28]. Also, due to normal karytoype in this patient, bracydactyly-mental retardation syndrome with deletion in chromose 2 is not considered. This deletion (MIM #600430) is often reffered to as Albright hereditary osteodystrophy-like syndrome. These patients with distal 2q deletions present with mild to moderate intellectual disabilities, hypotonia, obesity, short stature, and brachydactyly with short phalanges|(especially the third to fifth phalanges[brachydactyly type E], as seen in patients with Albright syndrome [29]. No dysmorphic features including thin, highly arched eyebrows, prominent forehead, depressed nasal bridge, hypoplastic alae nasi, prominent nasal septum, thin upper lip, and ear anomalies (which are part of brachydactyly-mental retardation syndrome) were not seen in our patient.

However the normal laboratory tests in our case, decline the diagnosis of PHP-Ia. Analysis of PHP-Ia and PPHP cases shows that individuals with isolated AHO and normal Gsα activity exists [22]. So identifying of such individuals may be useful to discover different genetic patterns in pseudohypoparathyroidism and pseudopseudohypoparathyroidism. Regarding to the reports by Nanclares et al. [5] Mantovani et al. [12] and Sussane Thiele et al. [21], these findings strengthen the need of new classifications of GNAS related disorders due to this discordance reported in this case and other similar cases described before.

The neuropsychiatric findings in our subject were compatible with Asperger syndrome diagnosis. Asperger syndrome is characterized by impairment in social interactions and restrictive-repetitive pattern of behaviors and interest. The syndrome belongs of Pervasive Developmental Disorders (PDD) or Autism spectrum disorders [30]. The aforementioned case with multiple medical complications had prominent neuropsychiatric manifestations such as; generalized Tonic-clonic seizures with Abnormal EEG, Stereotyped and repetitive motor mannerisms, severe impairment in social interactions, severe communication problems with family members, ritualistic behaviors and compulsions (washing and repeating), mild mental retardation. Warwick et al. [31] describes a case of a 23 years old man with obsessive-compulsive behavior and agitation, temporal lobe epilepsy (multiple generalized tonic-colonic seizures), some developmental delay, and markedly impaired social interactions toward family and at work with stereotyped and repetitive movements. He was diagnosed and treated as Asperger syndrome, that his symptoms are compatible with neuropsychiatric findings in our subject. In addition, the case of co-morbid Pseudohypoparathyroidism and Asperger syndrome manifestations has not been reported to now as we searched.

It will be important to identify patients in whom PHP (or PPHP subtype) is caused by novel GNAS findings or unusual clinical findings, as careful investigations of these findings will likely further our knowledge of this complex and this unique disorder. The most important point is that this case presented with unusual neurologic findings which has not been reported up to now.

## Conclusion

According to our findings this patient could not be categorized in any of PHP subgroups. Identifying of such individuals may be useful to discover different genetic patterns in pseudohypoparathyroidism and pseudopseudohypoparathyroidism. It is important to identify patients in whom PHP is caused by novel GNAS mutations, as careful investigations of these findings will likely further our knowledge of this complex and this unique disorder. In addition this case presented with unusual neuropsychiatric findings which has not been reported up to now.

## Consent

Written informed consent was obtained from the patient’s parents for publication of this Case report.

## Competing interest

The authors declare that they have no competing interest.

## Authors’ contributions

SHR and AS gave the idea and designed the study and do the Follow up of the patient. SHR also edited the article. ZJ does the follow up and drafted the manuscript. SMA investigates and drafted the neuropsychiatric findings’ section of the article. MMA does the genetic evaluations. All authors have read and approved content of the article.
